# Psychosocial Stress and Immunity—What Can We Learn From Pig Studies?

**DOI:** 10.3389/fnbeh.2018.00064

**Published:** 2018-04-03

**Authors:** Ulrike Gimsa, Margret Tuchscherer, Ellen Kanitz

**Affiliations:** Psychophysiology Unit, Institute of Behavioural Physiology, Leibniz Institute for Farm Animal Biology (FBN), Dummerstorf, Germany

**Keywords:** social stress, immunity, inflammation, neuroendocrine regulation, *Sus scrofa*

## Abstract

Psychosocial stress may impair immune functions and provoke the development of pathologies. The underlying communication between the brain and the immune system is being studied predominantly in rodents. However, pigs offer several advantages as preclinical models for humans because pigs are more similar to humans than rodents in many anatomical and physiological characteristics. Unlike in rodents, the main stress-induced glucocorticoid in humans and pigs is cortisol with a similar circadian rhythm. In this study, we summarize data on short-term and long-term effects of social stress in pigs for their immunity and neuroendocrine regulation with consequences for their health and well-being. As typical social stressors, regrouping, crowding, social isolation, and maternal deprivation have been studied. Psychosocial stress in pigs may affect various reactions of innate and adaptive immunity, such as leukocyte distribution, cytokine secretion, lymphocyte proliferation, and antibody production as well as immune responses to viral infection or vaccination. Furthermore, social stress may induce or promote gastrointestinal diseases through dysregulation of inflammatory processes. In piglets, psychosocial stress may also result in glucocorticoid resistance of lymphocytes, which has been discussed as a cause of allergic asthma in humans. Stress-related neuroendocrine alterations in the cortico-limbic structures, such as the prefrontal cortex, amygdala, hippocampus and hypothalamus, have been demonstrated in pigs at different ages. Based on these data, we propose using pigs as models for psychosocial stress in humans to study the mechanisms of brain-to-immune and immune-to-brain communication from the systemic level down to the cellular and subcellular levels.

## Introduction

There is growing evidence that psychosocial stress may affect the immune system in humans. Investigation of the underlying mechanisms of this brain-to-immune communication has been restricted by ethical considerations, which is why animal models are being employed. While rodent models are commonly used, they might not be ideal. Pigs are more similar to humans than rodents in many of their anatomical and physiological characteristics. Therefore, they are used as models for the study of cardiovascular diseases, in gastrointestinal, and pharmacological research as well as in xenotransplantation (Swindle and Smith, 1998; Ekser et al., 2015; Gonzalez et al., 2015; Wyns et al., 2015; Kalder et al., 2016; Wirthgen et al., 2016). Furthermore, the immune system of pigs is very similar to that of humans in terms of anatomy, function, and gene expression (Freeman et al., 2012; Meurens et al., 2012; Conrad and Johnson, 2015). It has been shown for several immune parameters that the degree of similarity between humans and pigs is significantly higher than the similarity between humans and mice (Dawson, 2011). In addition, pigs are used in neuroscience research because their brain anatomy and development are closer to those of humans than rodents' are (Holm and West, 1994; Lind et al., 2007).

Because of these similarities in immune system and neurobiology of pigs and humans, pigs provide a good model to study immunomodulation by psychosocial stress. It was shown that psychosocial stress in pigs may affect the immune system by alterations in cellular and humoral immune responses, vaccination reactions and glucocorticoid sensitivity. These stress-induced effects are regulated by neuroendocrine mechanisms, which involve the hypothalamic-pituitary-adrenal (HPA) axis, the sympathetic-adrenomedullary (SAM) axis, and the limbic system of the brain. There are indications that the HPA axis of pigs more closely resembles the human HPA axis than the axis in rats does. For example, piglets, like human neonates, have no ontogenetic phase in which they are hyporesponsive to stress or adrenocorticotropic hormone (ACTH) challenge (Kanitz et al., 1999), while neonatal rats hardly respond to stress from the age of postnatal day two until their second week of life (Sapolsky and Meaney, 1986). Nevertheless, there is expansive literature on early life stress in rodents reporting effects that are similar to those observed in humans (reviewed in: Bartolomucci, 2007; Hawkley et al., 2012). There are also species differences in circadian rhythms of glucocorticoids. While the circadian rhythm in pigs resembles the rhythm found in humans (Ruis et al., 1997), mice and rats have a circadian rhythm that is opposed to that of humans and pigs (Halberg et al., 1958; Jozsa et al., 2005).

Based on published data, we would like to demonstrate that pigs are excellent animal models to study the effects of psychosocial stress on immune functions and their underlying mechanisms.

## Social stress in pigs

Pigs are social animals that prefer living in groups with well-established social structures and dominance hierarchies. They strongly perceive social stress when these structures are disrupted. However, management procedures in pig husbandry often do not adequately consider the social needs of the animals and their social bonds. For example, repeated regrouping of pregnant sows represents a social stressor that may affect stress regulation and the immune systems of their offspring (Couret et al., 2009a,b; Otten et al., 2010; Sandercock et al., 2011), with stress in late pregnancy apparently being more relevant for the immune functions of offspring than stress in early pregnancy (Otten et al., 2015). Another important stressor in pigs is the weaning process, which besides environmental and nutritional changes has a strong psychosocial component. The piglets face a sudden and permanent maternal deprivation and regrouping with conspecifics (Campbell et al., 2013). To study the psychosocial stress of weaning, maternal deprivation and isolation have been thoroughly investigated in terms of stress-response regulation and immunomodulatory effects in different experimental models. Regrouping with unfamiliar conspecifics, which is typically performed several times in the lives of domestic pigs, disrupts established social structures. Increased basal salivary cortisol concentrations and a behavior indicating alertness were observed in repeatedly regrouped pigs (Coutellier et al., 2007). Loss of rank in a dominance hierarchy has been shown to be a severe stressor in pigs (Tuchscherer et al., 1998; Otten et al., 1999, 2002). Furthermore, it was shown that social disruption in pigs may affect immune responses in different ways (Hessing et al., 1994; Morrow-Tesch et al., 1994; Deguchi and Akuzawa, 1998; Tuchscherer et al., 1998; de Groot et al., 2001; Ruis et al., 2001; Rudine et al., 2007; Sutherland et al., 2007; Bacou et al., 2017).

The detailed information is summarized in Table 1 and will be discussed in the next chapter.

**Table 1:** Social stress effects on hypothalamic-pituitary-adrenocortical (HPA) axis, sympatho-adrenomedullary (SAM) axis, brain and immune system.

**Stressor**	**Treatment period/challenges**	**Effects on**				**References**
		**HPA activity**	**SAM activity**	**Brain neurotransmitters and cytokines**	**Immune system**	
**PRENATAL STRESS—EFFECTS ON OFFSPRING**						
Regrouping of pregnant sows	Twice a week during early gestation during late gestation	Adrenal weight ↓ CBG ↓ Adrenal weight ↑ Cell density cortex ↑ (weaning)	n.d. Adrenal weight ↑ Cell density medulla ↑ (weaning)	n.d. *hippocampus* 5-HIAA/5-HT ↑ (weaning) NA ↑ (relocation)	Immunization ↔ LPS challenge ↔ Leukocyte numbers ↓ lymphocyte proliferation ↑ TNF (LPS *in vitro*) ↓	Couret et al., 2009a,b; Otten et al., 2010
**POSTNATAL STRESS**						
Social isolation of piglets	PND 3 to 11 (2 h daily) LPS: PND 12 LPS: PND 56	Cortisol, ACTH ↑ CRH (*hypoth*.) ↓ CRH (*amyg*.) ↑ Cortisol, CBG, ACTH ↔ Cortisol, CBG, ACTH ↔	n.d.	IL-1β (*hippo*.) ↑ TNF (*hippo*.) ↓ IL-1β (*hippo*.) ↑	Lymphocyte proliferation ↓ TNF, IgG ↔ TNF ↓ IgG ↔	Kanitz et al., 2004; Tuchscherer et al., 2004, 2006
Social isolation of piglets	PND 7, 21, or 35 (4 h)	ACTH, cortisol ↑	n.d.	n.d.	TNF ↓ CD4+/CD8+ ratio ↓ IL-1β, IL-10 (LPS *in vitro*) ↓ Lymphocyte proliferation ↑ GC resistance ↑	Kanitz et al., 2009; Tuchscherer et al., 2009, 2010
Social isolation of piglets with conspecific	PND 7, 21, or 35 (4 h)	ACTH, cortisol, FCI ↓ CBG ↑	n.d.	n.d.	GC resistance ↓ IL-6, TNF (LPS *in vitro*) ↓	Kanitz et al., 2014, 2016; Tuchscherer et al., 2014, 2016
Social isolation of piglets followed by *E.coli* challenge	PND 7, 21, or 35 (4 h)	Cortisol ↑ MR mRNA, 11βHSD-1 mRNA (*spleen*) ↑	n.d.	IL-6 mRNA (*hypoth*.) ↑	IL-6 mRNA (*spleen*), TNF (*spleen*) ↑	Tuchscherer et al., 2018
Social isolation of gilts	PND 180 (5 days)	Cortisol ↑	n.d.	n.d.	Acute phase proteins ↑	Marco-Ramell et al., 2016
Weaning	PND 14 or 28	n.d.	n.d.	n.d.	Lymphocyte proliferation ↓	Blecha et al., 1983
Weaning	PND 28 LPS: PND 30	ACTH, cortisol ↑ CBG ↔	n.d.	n.d.	Lymphocyte proliferation ↓ (weaning)	Kanitz et al., 2002
Weaning	PND 19	CRH, cortisol ↑	n.d.	n.d.	Intestinal barrier ↓	Moeser et al., 2007a
Weaning	PND 15, 18, or 23	CRH, cortisol ↑ Age-dependent	n.d.	n.d.	Mast cell activity ↑ Age-dependent	Smith et al., 2010
Weaning	PND 19 or 28	CRH, cortisol ↑ (PND 19, 28) CRH (*mucosa*) ↑ (PND 19)	n.d.	n.d.	Intestinal barrier ↓ (PND 19) Mast cell activity ↑ (PND 19)	Moeser et al., 2007b
Regrouping of pairs of familiar piglets with unknown piglets	PND 33 (3 days)	Cortisol ↑	n.d.	n.d.	Lymphocyte proliferation ↔	Merlot et al., 2004
Regrouping	PND 45, 3 days after vaccination	Cortisol ↑	Noradrenaline ↑	n.d.	Vaccination ↓	de Groot et al., 2001
Regrouping	PND 42 (1 h)	Cortisol ↑	Noradrenaline ↑	n.d.	Leukocyte numbers ↑ TNF, IL-8 (LPS *in vitro*) ↓	Bacou et al., 2017
Regrouping	PND 84 (3 days)	Cortisol ↔	n.d.	n.d.	Lymphocyte proliferation (dominant pigs) ↑ IgG ↑	Tuchscherer et al., 1998
Regrouping	PND 64, 84, 91	Cortisol ↑	n.d.	n.d.	Lymphocyte proliferation ↓	Deguchi and Akuzawa, 1998
Regrouping, heat, crowding	PND 42 (14 days)	Cortisol ↓	n.d.	n.d.	Lymphocyte proliferation ↑ NK cell cytotoxicity ↑	Sutherland et al., 2006
Regrouping, crowding	PND 70 (7 days)	CRH (*mucosa*) ↑ cortisol ↑	n.d.	n.d.	Intestinal barrier ↓ TNF, IL-1β, IL-10 (*intestine*) ↑	Li et al., 2017
Social defeat	PND 70 (15 min)	ACTH, cortisol ↑	Adrenaline, noradrenaline ↑	n.d.	N/L ratio ↑	Ruis et al., 2001

*ACTH, adrenocorticotropic hormone; amyg, amygdala; CBG, corticoid-binding globulin; CRH, corticotropin-releasing hormone; E. coli, Escherichia coli; FCI, free cortisol index; GC, glucocorticoid; hippo, hippocampus; hypoth, hypothalamus; IL, interleukin; LPS, lipopolysaccharide; N/L ratio, neutrophil/lymphocyte ratio; n.d., not determined; PND, postnatal day; TNF, tumor necrosis factor*.

## Impact of social stress on immunity and inflammation

### Changes in innate immunity

Many studies have investigated social stress effects on leukocyte composition and innate immunity in pigs. Maternal social stress caused attenuated inflammatory responses to challenges in piglets (Sandercock et al., 2011) and reduced the number of circulating leukocytes as well as the CD4+/CD8+ T cell ratio in the offspring (Couret et al., 2009a). Weaning stress potentiated the neuroendocrine response to lipopolysaccharide (LPS) injection as a model for a bacterial infection and induced higher intensity sickness behavior, whereas plasma tumor necrosis factor (TNF) concentrations remained unaltered (Kanitz et al., 2002).

To explicitly study psychosocial stress, a repeated daily isolation procedure of piglets was performed. This isolation stressor diminished TNF increases after LPS challenge and enhanced signs of sickness, which resulted in a stronger relationship between duration of sickness symptoms and physiological measures (Tuchscherer et al., 2004, 2006). This outcome indicates that adaptive responses to immune challenges or disease are sensitized through early isolation. Piglets that experienced a single social isolation for 4 h displayed age-dependent decreases in plasma TNF concentrations and CD4+/CD8+ T cell ratios as well as diminished cytokine release in LPS-stimulated whole blood cultures (Tuchscherer et al., 2009). Furthermore, it was shown that the neutrophil/lymphocyte (N/L) ratio was increased in weaned piglets (Puppe et al., 1997) and in regrouped gilts after social defeat (Ruis et al., 2001).

Other studies document the effects of social stress on the capability to fight infections. Sows that were transferred from group to individual housing activated the innate immune system, as shown by the increased expression of haptoglobin and C-reactive protein (Marco-Ramell et al., 2016). Acutely stressed piglets, which had been moved to single cages on the day of weaning were less susceptible to a challenge infection with *Staphylococcus aureus* (Larson et al., 1985). It is possible that this isolation stress activated the innate immune system, as shown by Marco-Ramell et al. (2016), and led the pigs to be better equipped to handle the infection. A 1 h regrouping of pigs with unfamiliar conspecifics increased leukocyte numbers in the blood and decreased LPS-induced cytokine secretions in whole-blood assays (Bacou et al., 2017). In a recent study, a cross-sensitization between stress and the immune system has been found in piglets that were isolated once for 4 h and then repeatedly challenged with oral *Escherichia coli* infection. These piglets showed a stronger HPA and pro-inflammatory cytokine response to infection (Tuchscherer et al., 2018).

In general, studies in humans and animals have shown that chronic or repeated stress reduce immune responses whereas a single exposure to stress enhances immunity (Dhabhar, 2008), which is also supported by studies on innate immunity in pigs.

### Influences on adaptive immunity

#### Effects of pre- and postnatal social stress

To study social stress effects on adaptive immune responses, mitogen-induced lymphocyte proliferation has often been employed. For example, repeated regrouping of pregnant sows resulted in increased lymphocyte proliferation in response to mitogens in their offspring (Couret et al., 2009a). The authors speculate that prenatal stress affected the development of regulatory T cells, which control proliferative responses. Additionally, the glucocorticoid control of lymphocyte proliferation could have been affected through a phenomenon called glucocorticoid resistance (see section Glucocorticoid Resistance). In contrast, weaning of piglets suppressed mitogen-induced T lymphocyte proliferation (Blecha et al., 1983; Kanitz et al., 2002). Further, weaning followed by LPS challenge decreased IgG concentrations as a result of hyperactivation of the HPA axis (Kanitz et al., 2002).

In a model of repeated maternal deprivation/isolation, mitogen-induced T- and B-cell proliferation was inhibited 1 day but not 45 days after the isolation period, which indicated there was a transient effect (Kanitz et al., 2004). Furthermore, regrouping of unknown piglets resulted in agonistic behavior and long-lasting suppression of lymphocyte proliferation (Deguchi and Akuzawa, 1998). When regrouping was performed by introducing a pair of familiar piglets into a group of unknown piglets, an increase in salivary cortisol and behavioral changes indicated a stress response. The lymphocyte proliferation, however, was unchanged (Merlot et al., 2004). It could be assumed that regulatory T cells were affected. There are indications on the effects of psychosocial stress on regulatory T cells in humans and mice with contradictory results, which require further investigation (Wieck et al., 2013; Ronaldson et al., 2016; Schmidt et al., 2016).

#### Role of social status

Dominance hierarchies, which exist in many social species, are known to influence stress responses. In humans, the concept of rank is rarely applied. Instead, the socioeconomic status is used in psychoneuroimmunological studies as a variable (Sapolsky, 2005). Surely, the socioeconomic status is important in many aspects for human health. However, a subjectively perceived high social status and the threat of losing it have been shown to induce the strongest cortisol response to a laboratory stressor in humans of comparable socioeconomic status (Gruenewald et al., 2006). In pigs, a number of studies investigated the immunological effects of regrouping, which is accompanied by increasing or decreasing social status for some of the pigs. After regrouping of unfamiliar pigs, mitogen-induced proliferation was higher in dominant than in subordinate pigs. Additionally, it increased in dominant pigs and decreased in subordinate pigs with an increasing number of agonistic interactions, which indicates that the controllability and predictability are critical for the modulation of immune functions (Tuchscherer et al., 1998). In another study, dominant pigs had a higher cellular immune response and were less susceptible to a virus infection than subordinate pigs (Hessing et al., 1994). In contrast, vaccination responses have been shown to be suppressed in regrouped barrows, which resulted in stronger clinical symptoms after a challenge infection with pseudorabies virus (PRV). Here, the dominant pig seemed to be more affected than the subordinate (de Groot et al., 2001). Clearly more work is needed to understand the role of social status in immune responses and to explain these conflicting results.

In humans, bullying is a strong psychosocial stressor involving the social status of an individual. Björkqvist (2001) suggested that the terms “dominant” and “subordinate” in animal studies have their equivalent in “bully” and “victim” in human studies. However, human studies on immunomodulating effects of bullying are scarce (Haavet et al., 2004; Baldwin et al., 2018). While in humans, bullying is just as prevalent in females as in males, female aggression after regrouping is commonly low in mice and rats (Björkqvist, 2001) but has been demonstrated in adult female rats in specific social situations (Albert et al., 1988; Pittet et al., 2017). As aggression after regrouping can reliably be observed in female pigs (Stookey and Gonyou, 1994; D'Eath and Pickup, 2002), this species could serve as a good model for studying immunomodulatory effects of bullying in an experimental setting.

### Gastrointestinal changes

Psychosocial stress may also play a role in the onset and exacerbation of gastrointestinal diseases in humans and animals. The enteric nervous system is connected to the brain by parasympathetic and sympathetic pathways that are called the gut-brain axis (Bhatia and Tandon, 2005). While regrouping of piglets at an age of 32 days did not affect intestinal wall integrity (Koopmans et al., 2006), early weaning resulted in intestinal epithelial barrier dysfunction that was mediated by mast cell activation (Moeser et al., 2007a,b; Smith et al., 2010). It has been shown that the release of mast cell proteases induced by corticotropin-releasing hormone (CRH) is regulated by the enteric nervous system (Overman et al., 2012). Chronic regrouping/crowding stress in pigs impaired ileal and colonic barrier function, whereby the local pro-inflammatory cytokines TNF, IL-1β, and IL-8 were downregulated and the anti-inflammatory cytokine IL-10 was robustly upregulated (Li et al., 2017). These findings are in contrast to studies in rodents (Reber et al., 2008). Such immunosuppression may result in increased susceptibility to gastrointestinal infections, which has been observed in early-weaned piglets (McLamb et al., 2013). Furthermore, gastric ulcerations in slaughtering pigs have been associated with regrouping stress (Gottardo et al., 2017).

These examples show that the pig may also represent an important translational model for research into the influence of gut microbiota on the gut-brain axis, neuroimmunological processes and mood in the context of stress (Rhee et al., 2009; O'Mahony et al., 2015).

### Glucocorticoid resistance

It is known that acute psychosocial stress may result in glucocorticoid resistance of LPS-stimulated lymphocytes (Rohleder et al., 2003). A single episode of social isolation for 4 h reduced the sensitivity of mitogen-induced lymphocyte proliferation to cortisol in suckling piglets (Tuchscherer et al., 2010, 2014). However, social support from the presence of an age-matched conspecific, particularly a familiar piglet, attenuated the cortisol resistance (Tuchscherer et al., 2014, 2016). Although glucocorticoid resistance is probably an adaptive response that secures immune functions during short-term stress, it carries the risk of uncontrolled inflammation and has been discussed as a cause of allergic asthma in humans (Haczku and Panettieri, 2010).

For details regarding the stress models and immune effects, see Table 1.

## Neuroendocrine regulation of social stress

Stress activates neuroendocrine systems, which regulate behavior, metabolism, and immune reactions. The HPA axis is the major anatomical substrate of stress responses. Glucocorticoids, which are the end-product of the HPA activation, are important mediators of stress and exert their complex effects via corticosteroid receptors in specific brain areas, which modulate stress adaptation (de Kloet et al., 1998; De Kloet et al., 2005; Black, 2002). While physiological stressors directly activate the paraventricular nucleus of the hypothalamus, psychological stressors require higher processing involving cortico-limbic structures of the brain, such as the amygdala, hippocampus and prefrontal cortex (PFC) (Herman and Cullinan, 1997; Herman et al., 2005). These structures receive further input from the noradrenergic, serotonergic, and dopaminergic systems (Dunn, 1992; Kabbaj et al., 1996; de Laurentiis et al., 2002).

Changes in the limbic and the HPA systems have been observed in pig studies on regrouping, weaning and isolation stress. An elevated mRNA expression of the glucocorticoid metabolizing enzyme 11β-hydroxysteroid dehydrogenase (11β-HSD) 1 and an increased serotonin turnover after weaning has been shown in piglets of sows stressed by repeated regrouping during pregnancy. Additionally, neuronal activation in the hippocampus has been demonstrated by increased c-fos mRNA expression and noradrenaline concentrations in these piglets (Otten et al., 2010). Regrouping of sows during pregnancy may also affect the stress sensitivity of offspring according to hypothalamic and amygdala CRH mRNA expression (Jarvis et al., 2006). A previous study showed that the weaning process may reduce glucocorticoid receptor (GR) binding in the hippocampus and amygdala (Kanitz et al., 1998). At the level of gene expression, early weaning reduced mRNA expression of 11β-HSD1 and 2, GR and mineralocorticoid receptor (MR) in the hippocampus, while a brief social isolation for 15 min reduced expression of the same genes in the PFC (Poletto et al., 2006). In contrast, a single social isolation of piglets for 4 h caused elevated stress hormone release and open-field reactivity associated with increased mRNA expression of corticosteroid receptors and metabolizing enzymes in the hypothalamus and hippocampus, while in the amygdala, the MR mRNA expression was decreased. Interestingly, the elevated c-fos mRNA indicated neuronal activation in the hypothalamus and amygdala (Kanitz et al., 2009). Remarkably, social buffering by conspecifics during maternal deprivation attenuated or prevented stress-induced changes in the PFC, amygdala and hypothalamus (Kanitz et al., 2014, 2016). Further, it has been shown that repeated early social isolation resulted in elevated IL-1β concentrations in the hypothalamus and hippocampus as well as enhanced GR binding in the hippocampus. IL-1β drives sickness behavior and is one of the main mediators of brain-immune system communication. Accordingly, stressed piglets showed rather passive patterns of behavior, which can be interpreted as stress-induced sickness behavior or depression. While hypothalamic CRH was still reduced 7 weeks after isolation, it was increased in the amygdala (Kanitz et al., 2004). These data suggest that psychosocial stress may cause long-term effects in the brain-endocrine-immune system.

Effects of social stress on the expression of stress-related genes and the GR binding in the brains of pigs are summarized in Table 2.

**Table 2:** Expression of stress-related genes and glucocorticoid receptor (GR) binding in various brain regions.

**Stressor**	**Treatment period/Challenges**	**Hypothalamus**	**Hippocampus**	**Amygdala**	**Prefrontal cortex**	**References**
**PRENATAL STRESS—EFFECTS ON OFFSPRING**						
Regrouping of pregnant sows	Twice in mid- (MG) or late gestation (LG) Restraint (30 min) and isolation (1 h) at PND 60	CRH mRNA (MG)↑ CRH mRNA (LG) ↔ CRH mRNA↑	n.d.	CRH mRNA↑ CRH mRNA↑	n.d.	Jarvis et al., 2006
Regrouping of pregnant sows	Twice a week in late gestation	GR binding ↔	GR binding ↔ 11βHSD-1 mRNA ↑ (weaning) c-fos mRNA ↑ (relocation)	n.d.	n.d.	Otten et al., 2010
**POSTNATAL STRESS**						
Social isolation of piglets	PND 3 to 11 (2 h daily) LPS: PND 12 or 56	n.d.	GR binding ↑ GR binding ↓	n.d.	n.d.	Kanitz et al., 2004; Tuchscherer et al., 2004
Social isolation of piglets	PND 7, 21 or 35 (4 h)	GR mRNA, 11βHSD-1 mRNA, c-fos mRNA ↑	GR mRNA ↔ 11βHSD-1 mRNA ↑	c-fos mRNA ↑	n.d.	Kanitz et al., 2009
Social isolation of piglets with conspecific	PND 7, 21 or 35 (4 h)	MR/GR mRNA ratio ↑ 11β-HSD2 mRNA ↔	MR/GR mRNA ratio, 11β-HSD2 mRNA, c-fos mRNA ↔	MR/GR mRNA ratio ↑ 11β-HSD2 mRNA, c-fos mRNA ↓	MR/GR mRNA ratio ↑ 11β-HSD2 mRNA ↓ c-fos mRNA ↔	Kanitz et al., 2014, 2016
Social isolation of piglets followed by *E. coli* challenge	PND 7, 21 or 35 (4 h)	11β-HSD1 mRNA, 11β-HSD2 mRNA, IL-6 mRNA ↑	n.d.	n.d.	GR mRNA, 11βHSD-1 mRNA, 11β-HSD2 mRNA ↑	Tuchscherer et al., 2018
Weaning followed by social isolation	PND 10 Isolation: PND 12 or 23 (15 min)	n.d.	11β-HSD1 mRNA, 11β-HSD2 mRNA, GR mRNA, MR mRNA ↓ 11β-HSD1 mRNA, 11β-HSD2 mRNA, GR mRNA, MR mRNA ↔	n.d.	11β-HSD1 mRNA, 11β-HSD2 mRNA, GR mRNA, MR mRNA ↔ 11β-HSD1 mRNA, 11β-HSD2 mRNA, GR mRNA, MR mRNA ↓	Poletto et al., 2006
Weaning	PND 35	GR binding ↔	GR binding ↓	GR binding ↓	n.d.	Kanitz et al., 1998

*11β-HSD, 11β-hydroxysteroid dehydrogenase; CRH, corticotropin-releasing hormone; E. coli, Escherichia coli; IL, interleukin; LPS, lipopolysaccharide; MR, mineralocorticoid receptor; n.d., not determined; PND, postnatal day*.

## Limitations of pig models

It should be noted that psychoneuroimmunological studies in pigs are limited for logistic and scientific reasons. Pigs are large outbred animals with a comparatively low breeding rate. With respect to analytical methods, certain impediments exist: (1) the variety of specific antibodies for pig proteins is limited; (2) immunohistochemical studies on pig brains are difficult because pig brains cannot be easily perfused; (3) as of yet, functional MRI can only be performed in anesthetized pigs, which precludes using it for the investigation of emotional processing; (4) there are only a few transgenic pig models to study the role of specific genes (Aigner et al., 2010; Holm et al., 2016). However, based on the increased acceptance of the pig as a valuable model for biomedical research, these problems will be overcome in the near future.

## Conclusions and future directions

Psychosocial stress in pigs has often been studied in experimental setups that are similar to common practices in animal husbandry. Although these stressors usually do not have equivalents in human life, they may reflect the moderate intensity of psychosocial stress in the human society better than some of the rodent stress models. Interestingly, pig studies show that social rank and agonistic interactions to achieve or defend rank have a substantial influence on immune responses. The findings strongly suggest investigating immunomodulatory social stress effects in humans based on their social rank. The main advantage of pig models is to gather information, which is not available from human studies due to ethical reasons. For example, prenatal and early postnatal stress models in pigs may reveal developmental alterations in neuroendocrine stress regulation and immunity with relevance for humans. Present limitations of pig models are counterbalanced by the opportunity to perform complex investigations of behavioral, neuroendocrine, and immunological interactions in a species, which is not only physiologically highly similar to humans but also in its emotionality and social behavior. Despite of these advantages, we would like to emphasize that both, rodent and pig models have their justifications. Combining their specific strengths may boost psychoneuroimmunological research into social stress in humans.

## Author contributions

UG, MT, and EK contributed to the conception and the design of the perspective. UG wrote the manuscript. MT and EK revised it critically for important intellectual content.

### Conflict of interest statement

The authors declare that the research was conducted in the absence of any commercial or financial relationships that could be construed as a potential conflict of interest.
